# What Galvanic Vestibular Stimulation Actually Activates

**DOI:** 10.3389/fneur.2012.00117

**Published:** 2012-07-20

**Authors:** Ian S. Curthoys, Hamish Gavin MacDougall

**Affiliations:** ^1^Vestibular Research Laboratory, School of Psychology, University of SydneyNSW, Australia

**Keywords:** vestibular, otolith, semicircular canal, nystagmus

## Abstract

In a recent paper in Frontiers Cohen et al. (2012) asked “What does galvanic vestibular stimulation actually activate?” and concluded that galvanic vestibular stimulation (GVS) causes predominantly otolithic behavioral responses. In this Perspective paper we show that such a conclusion does not follow from the evidence. The evidence from neurophysiology is very clear: galvanic stimulation activates primary otolithic neurons as well as primary semicircular canal neurons (Kim and Curthoys, 2004). Irregular neurons are activated at lower currents. The answer to what behavior is activated depends on what is measured and how it is measured, including not just technical details, such as the frame rate of video, but the exact experimental context in which the measurement took place (visual fixation vs total darkness). Both canal and otolith dependent responses are activated by GVS.

## Introduction

A recent paper in Frontiers in Neuro-otology by Holstein et al. (2012) described the regions in rat brain activated by galvanic vestibular stimulation (GVS) which is small direct current (DC) or slowly varying DC applied to the mastoids. GVS is now being used widely for human studies of vestibular function and so the evidence from animal studies as to the neural regions activated and the mechanism by which GVS generates behavioral responses is important. The method Holstein et al. (2012) used for showing brain regions was c-Fos labeling, a method of imaging active regions of the brain, with its own idiosyncrasies. The results had one unusual feature: that some brain regions, known by previous anatomical and physiological studies to be involved in generating vestibular-evoked behavioral responses, did not show c-Fos labeling and so appeared to be inactive during GVS. Holstein et al. explained this negative result by noting that studies in other modalities had shown that c-Fos does not necessarily show all regions activated. They explained these negative results thus:

Since c-Fos protein is not expressed in neurons that are tonically inhibited (Chan and Sawchenko, 1994), and since many vestibulo-ocular neurons receive substantial direct inhibition from cerebellar Purkinje cells and/or vestibular commissural fibers (Holstein et al., 1999; for reviews, see Holstein, 2012; Highstein and Holstein, 2006), it would be surprising if the magnocellular medial vestibular nucleus (MVN) neurons involved in vestibulo-ocular reflex pathways accumulated c-Fos protein. Similarly, vestibulo-spinal and vestibulo-colic neurons did not appear to express c-Fos in our study. This is most likely due to intrinsic cytological differences between sensory and motor pathway neurons, since c-Fos is primarily activated by sensory stimuli, and is rarely observed in brainstem neurons involved in motor pathways (Chan and Sawchenko, 1994). Thus, we would not expect the vestibulo-ocular, -spinal, and -colic motor neurons of the VNC to display c-Fos stain, even though many of these cells are at least transiently activated by sGVS (Holstein et al., 2012, p. 9).

This would appear to be a reasonable explanation of the apparent lack of activation of regions of vestibular nuclei in response to a vestibular stimulus.

In a companion paper Cohen et al. (2012) used the negative results of Holstein et al. (2012) to put forward a different idea about the mechanism of galvanic vestibular responses. The title of this Opinion paper “What does GVS actually activate?” is ambiguous, since that question can be asked of receptor mechanisms at the periphery or of responses generated (“activated”) by the galvanic stimulus. This Opinion paper needs careful analysis because it has far-reaching implications for understanding vestibular processing. Cohen et al. (2012) answered the question they posed in the title by concluding on the basis of the Holstein et al. evidence and other behavioral data from GVS stimulation that “despite this non-selective activation, it appears that only otolith-related behavioral responses are induced” (p. 1). In this Perspective paper we show that such a conclusion does not follow from the evidence. We address the argument of Cohen et al. (2012) and clarify questions of definition and some relevant matters, since much of the force of their argument relies on physiological and behavioral data obtained in our laboratory.

## Terms

In most vestibular studies on human subjects GVS is applied through very large surface electrodes (600–900 mm^2^) placed over the mastoid with generous electrode paste to ensure good skin contact. The usual maximum current is about 5 mA since higher currents or smaller electrodes cause strange skin sensations and risk burning the subject’s skin. We distinguish this from the methods used by Cohen and Suzuki in a landmark series of papers, who used high-frequency electrical stimulation by very fine bipolar stainless steel electrodes implanted onto the axons from the ampullae of cats and monkeys to demonstrate the direction of eye movements from stimulation of isolated nerves from each semicircular canal and the utricular macula (beginning with Cohen and Suzuki, 1963; see also Cohen et al., 1964; Suzuki et al., 1969). In contrast GVS is usually a weak current which probably acts at the spike trigger zone of vestibular afferents (Goldberg et al., 1982, 1984), rather than causing membrane depolarization: maintained GVS generates a maintained series of action potentials (which adapt) during the DC stimulus (Kim and Curthoys, 2004).

## Evidence from Physiological Recordings in the Vestibular Periphery

Kim and Curthoys conducted experiments to address the question of what GVS activates in the vestibular periphery by recording single primary vestibular afferents in Scarpa’s ganglion in guinea pigs and determining the threshold of these neurons for activation by GVS (2004). In most of these experiments the stimulating electrodes for delivering GVS were syringe needles inserted in the ball of the tensor tympani muscle very close to the receptors in the vestibular labyrinth. The currents needed to activate individual vestibular afferents at threshold using these electrodes were very small – in some cases as small as 5 microamps (Kim and Curthoys, 2004). In some experiments surface electrodes were used on the guinea pig mastoid, analogous to the surface electrodes in human GVS studies, and with these electrodes, much higher currents were needed (by about a factor of 10 or more) to activate neurons compared to the current via the needle electrodes. Importantly in both stimulation paradigms, galvanic stimulation was found to activate afferent neurons from all vestibular endorgans about equally. These results confirmed an earlier study by Goldberg et al. (1984).

## The Responses to GVS in Human Subjects

In alert humans and guinea pigs GVS elicits oculomotor and postural responses. However Cohen et al. (2012) wrote: that in response to mastoid GVS, human subjects “… do not display ocular nystagmus which would occur if the semicircular canals were continuously stimulated” (p. 1). Published evidence shows that this statement is not correct. We have shown that GVS does induce nystagmus with both horizontal and torsional components when it is measured in darkness with adequate sampling rates (MacDougall et al., 2002, 2003, 2005; see also Vailleau et al., 2011). The statement by Cohen et al. (2012) above referred to early results from our laboratory (Watson et al., 1998), in which we measured oculomotor responses in human subjects to GVS via mastoid stimulation under very limited conditions. The Watson et al. (1998) paper was the start of a long series of experiments in which there were major technical advances in the course of the experiments. Initially (Watson et al., 1998) we used a very low sampling rate (2 Hz) for our video acquisition system to allow us to use the then newly developed algorithms for measuring torsion accurately by video, and for that same practical reason there was always a fixation light present – to suppress eye movements of any kind which interfered with the torsion algorithms. Under these conditions with very low sampling rate and a visual fixation point horizontal nystagmus was rarely observed, as we noted in that paper, but in later experiments as our algorithms improved and the video frame rate increased, the experimental conditions were improved – measures were made at high frame rates in total darkness with no fixation light present – and in these conditions the horizontal (and torsional) nystagmus was very clear. In fact the GVS-induced nystagmus and its suppression by vision was a major point of two papers (MacDougall et al., 2002, 2003).

**Figure 1:**
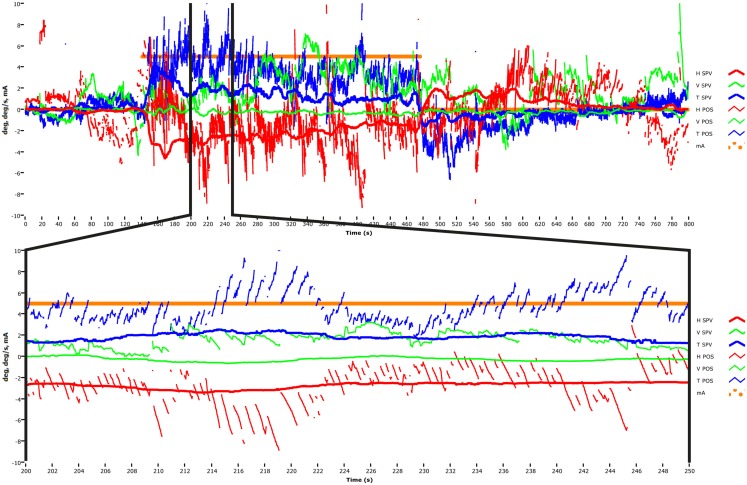
**Upper panel: time series of the horizontal, vertical, and torsional eye position and eye velocity recorded by video acquisition to a 5 mA step of galvanic current applied between large surface electrodes over the two mastoids in a healthy subject (methods in MacDougall et al., 2002)**. At the onset of the GVS there is a vigorous horizontal nystagmus (red traces) and a vigorous torsional nystagmus (blue traces). There is very little vertical nystagmus (green traces). Lower panel: one section of the long time series is magnified so each beat of horizontal and torsional nystagmus is clearly visible. This data was obtained at 30 Hz sampling rate in total darkness and it shows that small value GVS does elicit low velocity horizontal nystagmus.

In these publications we plotted slow-phase eye velocity (SPV) of GVS-induced horizontal and torsional nystagmus in response to various levels of GVS stimulation, in light and in total darkness. These data were desaccaded eye position data which had been differentiated to yield the SPV. A published raw data figure (MacDougall et al., 2005, p. 505) shows the horizontal nystagmus very clearly, and another such (unpublished) raw data figure from that study is shown here (Figure 1) as a representative example to show that horizontal nystagmus really is produced by GVS. This is an unpublished record with a 30 Hz sampling rate from H. G. MacDougall’s PhD thesis research (MacDougall et al., 2003). All the technical details for the figures are given in MacDougall et al. (2003). Figure 1 shows that in darkness, in response to a 5 mA step of galvanic current between the mastoids the subject had a small but clear horizontal nystagmus (of about 5°/s with slow phases toward the anode). In light that nystagmus disappeared due to the well known visual suppression of modest horizontal nystagmus by a visual fixation point (Baloh et al., 1975; Zee, 1977; Halmagyi and Gresty, 1979). It is not a strong nystagmus (the peak SPV was usually less than about 10°/s) and the SPV velocity depends on the galvanic current – we found a relationship of about 1°/s/mA. So at the 5 mA typically used, the peak SPV of the horizontal nystagmus is only about 5°/s maximum. The conclusion of MacDougall et al. (2003) was that there was a highly linear relationship between peak SPV and galvanic current strength – even at very low GVS intensities, horizontal nystagmus was induced, but with a very small SPV. The video method has very good signal-to-noise ratio and so even these very small eye velocities were easily detectable.

In his later reply to Dr. Colebatch (2012), Dr. Cohen et al. (2012) acknowledges that GVS does cause horizontal nystagmus, but asserts that this occurs because the stimulus current levels used by MacDougall et al. were so large that the nystagmus could not be suppressed. As we have shown above, this is not correct. In fact the effective magnitude of GVS current in the MacDougall studies was very small (5 mA maximum at the mastoid and in light of the reduction found in guinea pigs we can reasonably conclude that the current at the vestibular receptor regions was reduced by a factor of at least 10 and probably more), and delivered over a very wide area using large-surface-area electrodes. At these small currents the SPV of the nystagmus was correspondingly small (about 5°/s max). Most healthy subjects can suppress a nystagmus of a few deg/s (Leigh and Zee, 1983) in, e.g., caloric and rotational nystagmus, and our own published data themselves from testing with and without vision, show that subjects easily suppress the GVS induced nystagmus if vision is present (see MacDougall et al., 2002, Figure 3).

## Conditions for Detecting Nystagmus to GVS

(1) Visual fixation must be absent. For galvanically induced nystagmus, just as for caloric and rotational nystagmus, if a fixation point is present, the nystagmus is suppressed by vision partially or totally depending on stimulus strength.
(2) The sampling rate of the data acquisition must be sufficiently high. If the sampling rate is too low (e.g., as low as 2 Hz in the study of Watson et al., 1998), then the quick phases of nystagmus will not be detected, and so it will appear that no nystagmus is present.

## The Importance of Context in Interpreting Response to GVS

The clear evidence of visual suppression of GVS-induced nystagmus goes to the heart of the arguments of Cohen et al. (2012). They argue from what they consider is the absent behavioral response of the horizontal canals – nystagmus – to speculate about what GVS is activating. In addition, recently Reynolds and Osler (2012) have reviewed the evidence showing that GVS also induces sensations of rotation in human subjects. As the above shows, the presence of some responses to GVS depends heavily on context as well as on the galvanic stimulus. By the word context we mean variables such as the presence of vision. The example from MacDougall et al. (2002) is especially informative here, since in the studies of MacDougall et al. the context was varied from full light to darkness while the GVS stimulus (and presumably the peripheral activation) remained the same, but the results were completely different. If one simply used the oculomotor response, one would be led to the conclusion that when vision was present the galvanic stimulation was not stimulating the horizontal canals! It is very clear that visual suppression of the vestibular-evoked response is the reason for that difference. That suppression is mediated by cerebellar inhibition onto neurons in the vestibular nuclei. The point is that changed context leads to changed behavior, whereas the activation of the peripheral vestibular sensory regions remains, almost certainly, unchanged. Other contextual effects such as head position can alter the behavioral responses, and these alterations are almost certainly mediated by neural changes at the vestibular nuclei.

The importance of context is further shown by considering the vertical nystagmus to GVS. In healthy subjects why is there almost no vertical nystagmus (Figure 1), when the physiological evidence is that primary afferents from the vertical canals are activated by GVS? It is usually argued that simultaneous stimulation of the anterior and posterior canals in the one labyrinth will act to produce oppositely directed nystagmus, and so the responses due to simultaneous stimulation of both canals by GVS in a healthy person would be expected to cancel. That idea of cancelation was confirmed by the oculomotor response to GVS of a patient, independently diagnosed as having inferior vestibular neuritis, so that the nerve from the posterior canal was dysfunctional, whereas the nerve from the anterior canal was functional. In this patient, unlike in healthy subjects, GVS did elicit clear vertical nystagmus in accord with the cancelation prediction (MacDougall et al., 2005). Once again from the absence of a response in healthy subjects (the absence of vertical nystagmus to GVS in this case) it is not possible to conclude that activation is non-existent. Absence of evidence is not evidence of absence.

## Questions Raised

Although the published data are not in accord with the opinion expressed by Cohen et al. (2012), some interesting ideas flow from that opinion. Are neural mechanisms of visual suppression equivalent for canal and otolith responses? There is evidence that some otolith-ocular responses can be suppressed by vision (Gianna et al., 2000) just as canal ocular responses can be. In the study of Holstein et al. (2012), if light was present during the GVS stimulation then it probably suppressed nystagmus and would so act to produce the negative result found by Holstein et al.

## Equivalence

Cohen et al. (2012) raise the question: does GVS primarily or exclusively activate the otolith system, or does it activate both the otolith and semicircular canal systems equivalently. But how would it be possible to measure “equivalent” activation? Presumably the outcome of uniform peripheral activation is going to depend on many factors, such as (a) relative numbers of afferents from each sensory region (Lopez et al., 2005); (b) numbers of irregular afferents, since they have a low threshold for galvanic activation (Goldberg et al., 1984; Kim and Curthoys, 2004); (c) the relative “potency” or saliency of the projections at the vestibular nuclei; and (d) as we have shown, the conditions under which the elicited response occurs are especially important.

## Irony

We note the irony that the Opinion piece by Cohen et al. highlights the otolith contribution to galvanic induced nystagmus and assumes the canals make little contribution, whereas another account of galvanic induced responses highlights the canal contribution and largely ignores any otolith contribution (Fitzpatrick and Day, 2004). The physiological evidence is that GVS activates afferents from all sensory regions but the contribution of the otoliths in response to GVS was largely ignored in the model of Fitzpatrick and Day (2004). We think both of these extreme positions are untenable. The possibility of putting forward such diametrically opposite positions for the mechanism of the same GVS emphasizes the point of the present paper – that there are major problems of interpreting complex behavioral responses to such complex stimuli. Simply ignoring the demonstrated activation of otolithic afferents or canal afferents in explaining an observed response would seem an unproductive way of proceeding. The simple fact is that *both* canals and otolith primary afferents are activated and depending on circumstances, both canal and otolith behavioral responses are generated so any account which favors only canals or only otoliths is suspect.

## Conclusion

The result of simultaneous stimulation of all peripheral vestibular afferents is, as one would expect, complex. GVS applied to the mastoids of human subjects produces complex oculomotor, perceptual, and postural responses. But exactly what responses occur, depends on many factors, including the stimulus, the electrodes, the context, and how the responses are measured. It is a major challenge to try to interpret the complex responses to such a complex stimulus in terms of contributions from each vestibular endorgan; in part because to our knowledge there is no way of equating canal and otolith stimulation. GVS has a useful role in vestibular investigations. The presence of a response to GVS demonstrates there must be afferent fibers from vestibular receptors present (MacDougall et al., 2005). This can be valuable information in understanding patient complaints, for example after surgery for acoustic schwannoma.

## Conflict of Interest Statement

The authors are unpaid consultants to Otometrics.

## Abbreviations

GVS, galvanic vestibular stimulation; MVN, medial vestibular nucleus; sGVS, sinusoidal galvanic stimulation; SPV, slow-phase eye velocity; VNC, vestibular nuclear complex.
